# Supplementary material for: HSPA1L Enhances Cancer Stem Cell-Like Properties by Activating IGF1Rβ and Regulating β-Catenin Transcription
Source: Int J Mol Sci. 2020 Sep 22;21(18):6957. doi: 10.3390/ijms21186957 (PMC7555772; doi:10.3390/ijms21186957)
Supplement: Supplementary file 1 [file ijms-21-06957-s001.pdf]

Figure S1.

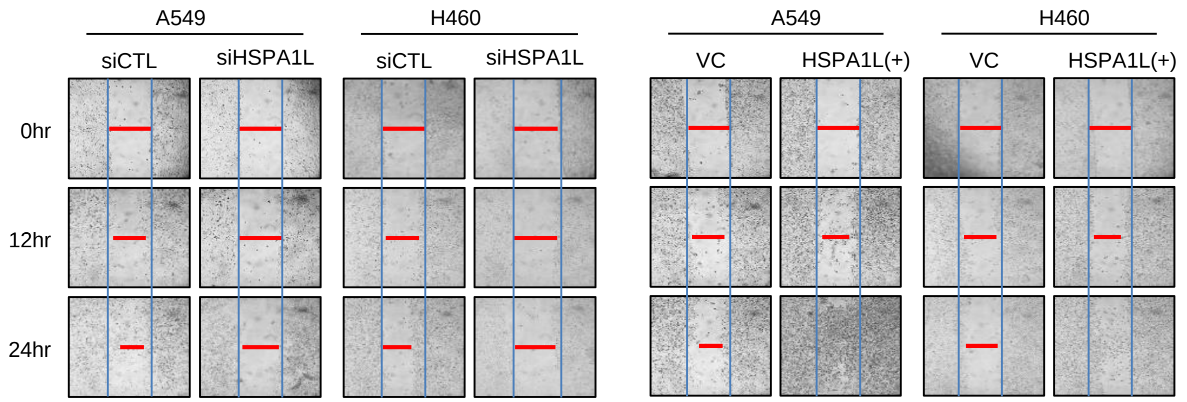

Figure S1. Wound-healing assay of A549 and H460 lung cancer cells transfected with siRNA targeting HSPA1L and pcDNA-HSPA1L.

Figure S2.

```
-995  AGAAAAATCCCCACAAATAAATCTATTGATACCTAGTGACAAGTGGAACCAGATAAAAAT  -936
      NFκB
-935  GGAATCTATAAGAATTAACCTAATTGACAGCGCTCTGGAGCTAATCCATTTCCATTAGTT  -876
-875  ATTTGTTCACAGTAGGTACTCCTAAGGACTTGTTGAATTGCGGGCTTGGCGCCCGTTCTA  -816
-815  CGGGAGAGTTGCACAGCCTTCGTGAGTGGGGACAGAAGGCGGCTCGGCCCGGTGATTCAG  -756
                                                                AP1
-755  GTCGAAATTCAAGCTGAACAGCCTGCTGAGAGGTGGGATCCACCATCCGGACAGTGGGGG  -696
-695  GCTTTGGGGGTGCTGTGAGACTGGGCTGCGACCCAGGTCCAGCAGGGAGTGTGCGGCACA  -636
-635  GACCACAAGGTCGGCGAGGCCCCCTAACCCGCGCCCGGCCGGGAACCCGCAGACCAGCGA  -576
                 AP2
-575  CGGGGCAGCTGCGGGGCCAGGAGCGCCCCAAGACGGGCGGGCGCTGAACCCGAGCCCCTG  -516
                             SP1/AP2      SP1
-515  TCGCCGCCCTGGGCCCGAACTTCCGCCCTCCCAGGACCTGTCCCGGCCGCCCCGAGCGGT  -456
         SP1/AP2                                          SP1
-455  ACTCGAAGGCCGGGGCCGAGATGCCACCTTCCGCAGGCCGCGGGAAAGGCGCGCCGAGTC  -396
                                        AP2
-395  CTGCAGCTGCTCTCCCGGTTCGGGAAACGCGCGGGGCGGGGGCGTCGGGCTTGGGACAGG  -336
                                          AP2/SP1/EGR1
-335  GGAGGATACCAGGGCCACCTTCCCCAACCCAGGCCGCGGGGGCCCGGCCTCCCCGATGCA  -276
                              AP2                       AP2
-275  GACCACAGCGCCCTCACGGGCTGCCCTCAGGCCGCGCAGCGGGCAGCCGCCAGCCGTCAC  -216
                          AP2                          AP2
-215  CCCGGGGAGCGTCCGTGGGGTGCCCAGGCACCCCACCCCGGCCCGGGGCGCTCAGACGGC  -156
                         AP2                       AP2
-155  AGCAGACTGCTGGGCGGCGCGGGGACTACTTTCCACCGCCCCCTCGCGCCCCGCCCCTTG  - 96
                SP1                        SP1            SP1
- 95  TCCTCGCGCGGCGGAACGCTCCGCGCTGCGCCGGTGGCGGCAGGATACAGCGGCTTCTGC  - 36
                                                    AP2
                                          +1
- 35  GCGACTTATAAGAGCTCCTTGTGCGGCGCCATTTTAAGCCTCTCGGTCTGTGGCAGCAGC  + 25
         TATA-BOX                         ↑
```

Figure S2. Nucleotide sequence of the β-cateinin promoter site. The transcriptional start site (TSS) is indicated by a vertical arrow and referred to as position +1. The putative TATA-box and other element names are given below the sequences.

Figure S3.

-995 AGAAAAATCCCCACAAATAAATCTATTGATACCTAGTGACAAGTGGAACCAGATAAAAAT -936

-935 GGAATCTATAAGAATTAACCTAATTGACAGCGCTCTGGAGCTAATCCATTTCCATTAGTT -876

-875 ATTTGTTCACAGTAGGTACTCCTAAGGACTTGTTGAATTGCGGGCTTGGCGCCCGTTCTA -816

-815 CGGGAGAGTTGCACAGCCTTCGTGAGTGGGGACAGAAGGCGGCTCGGCCCGGTGATTCAG -756

-755 GTCGAAATTCAAGCTGAACAGCCTGCTGAGAGGTGGGATCCACCATCCGGACAGTGGGGG -696

-695 GCTTTGGGGGTGCTGTGAGACTGGGCTGCGACCCAGGTCCAGCAGGGAGTGTGCGGCACA -636

-635 GACCACAAGGTCGGCGAGGCCCCCTAACCCGCGCCCGGCCGGGAACCCGCAGACCAGCGA -576

-575 CGGGGCAGCTGCGGGGCCAGGAGCGCCCCAAGACGGGCGGGCGCTGAACCCGAGCCCCTG -516

-515 TCGCCGCCCTGGGCCCGAACTTCCGCCCTCCCAGGACCTGTCCCGGCCGCCCCGAGCGGT -456

-455 ACTCGAAGGCCGGGGCCGAGATGCCACCTTCCGCAGGCCGCGGGAAAGGCGCGCCGAGTC -396

-395 CTGCAGCTGCTCTCCCGGTTCGGGAAACGCGCGGGGCGGGGGCGTCGGGCTTGGGACAGG -336

P3 -335 GGAGGATACCAGGGCCACCTTCCCCAACCCAGGCCGCGGGGGCCCGGCCTCCCCGATGCA -276

-275 GACCACAGCGCCCTCACGGGCTGCCCTCAGGCCGCGCAGCGGGCAGCCGCCAGCCGTCAC -216

-215 CCCGGGGAGCGTCCGTGGGGTGCCCAGGCACCCCACCCCGGCCCGGGGCGCTCAGACGGC -156 P2

-155 AGCAGACTGCTGGGCGGCGCGGGGACTACTTTCCACCGCCCCCTCGCGCCCCGCCCCTTG - 96

P1 - 95 TCCTCGCGCGGCGGAACGCTCCGCGCTGCGCCGGTGGCGGCAGGATACAGCGGCTTCTGC - 36

- 35 GCGACTTATAAGAGCTCCTTGTGCGGCGCCATTTTAAGCCTCTCGGTCTGTGGCAGCAGC + 25

+ 26 GTTGGCCCGGCCCCGGGAGCGGAGAGCGAGGGGAGGCGGAGACGGAGGAAGGTCTGAGGA + 85

P4 + 86 GCAGCTTCAGTCCCCGCCGAGCCGCCACCGCAGGTCGAGGACGGTCGGACTCCCGCGGCG +145

+146 GGAGGAGCCTGTTCCCCTGAGGTGCTTGGGCGCTCCTTTCCTTATCCTTCCGGGGCTGCT +205

+206 CCCGCTTCCTCTCGGAGCCAAACTTCGTAGCAGGCGCGCGGTCCGGACGGCGGGCTGGGC +265

+266 GCAGCCGGGAGGCCTGGGGTTGGGAGCGGGGAGCTCAGGTGGGGGACGGTGAGGGTGGCC +325

+326 CGCGCCCGGGACGCGGAGGGCGGCGGCCGGGCCCGGGTTCCGGTCGCGCTGCCTCTCTGG +385

+386 GGCCCTGGGGGCATCGCTTGCGGGGAGGGGGCGCCGCGGGGGCGCGTACAGGAGCCCGGA +445

+446 TGGCAGGCGGGGTGGGGGTGGGGGTTGGGGGTCTGTGGTTTCCGTCCGGGGCTCTGGCCT +505

+506 TGGCCGAGTTTGGGGGAGGGACCCGGTGCCTCGGGATGCGCCGGGCCCTGGGTGGGGGGC +565

+566 GGGGTGGGGACGGGGGGCTCCGCCTTCTCAGCTCTTGCGGCGAGTTGGGGTTCGGGCGCT +625

+626 GAGGCAGAGACGCCACCCTAAGTCCCATCAGTCCTG

Figure S3. Nucleotide sequence of the four different predicted specific promoter regions to which HSPA1L binds in β–catenin. Promoter primer sequences are listed in Table1.
